# A roadmap for urban evolutionary ecology

**DOI:** 10.1111/eva.12734

**Published:** 2018-12-14

**Authors:** L. Ruth Rivkin, James S. Santangelo, Marina Alberti, Myla F. J. Aronson, Charlotte W. de Keyzer, Sarah E. Diamond, Marie‐Josée Fortin, Lauren J. Frazee, Amanda J. Gorton, Andrew P. Hendry, Yang Liu, Jonathan B. Losos, J. Scott MacIvor, Ryan A. Martin, Mark J. McDonnell, Lindsay S. Miles, Jason Munshi‐South, Robert W. Ness, Amy E. M. Newman, Mason R. Stothart, Panagiotis Theodorou, Ken A. Thompson, Brian C. Verrelli, Andrew Whitehead, Kristin M. Winchell, Marc T. J. Johnson

**Affiliations:** ^1^ Department of Ecology and Evolutionary Biology University of Toronto Toronto Ontario Canada; ^2^ Department of Biology University of Toronto Mississauga Mississauga Ontario Canada; ^3^ Centre for Urban Environments University of Toronto Mississauga Mississauga Ontario Canada; ^4^ Department of Urban Design and Planning University of Washington Seattle Washington; ^5^ Department of Ecology, Evolution and Natural Resources Rutgers, The State University of New Jersey New Brunswick New Jersey; ^6^ Department of Biology Case Western Reserve University Cleveland Ohio; ^7^ Department of Ecology, Evolution, and Behavior University of Minnesota St. Paul Minnesota; ^8^ Redpath Museum and Department of Biology McGill University Montreal Quebec Canada; ^9^ Department of Forest and Conservation Sciences University of British Columbia Vancouver British Columbia Canada; ^10^ Museum of Comparative Zoology and Department of Organismic and Evolutionary Biology Harvard University Cambridge Massachusetts; ^11^ Department of Biology Washington University in Saint Louis Saint Louis Missouri; ^12^ Department of Biological Sciences University of Toronto Scarborough Toronto Ontario Canada; ^13^ School of BioSciences The University of Melbourne Melbourne Victoria Australia; ^14^ Integrative Life Sciences Doctoral Program Virginia Commonwealth University Richmond Virginia; ^15^ Louis Calder Center—Biological Field Station Fordham University Armonk New York; ^16^ Department of Integrative Biology University of Guelph Guelph Ontario Canada; ^17^ General Zoology, Institute of Biology Martin Luther University Halle‐Wittenberg Halle (Saale) Germany; ^18^ Biodiversity Research Centre and Department of Zoology University of British Columbia British Columbia Canada; ^19^ Center for Life Sciences Education Virginia Commonwealth University Richmond Virginia; ^20^ Department of Biology Virginia Commonwealth University Richmond Virginia; ^21^ Department of Environmental Toxicology University of California Davis Davis California; ^22^ Department of Biology University of Massachusetts Boston Boston Massachusetts

**Keywords:** community engagement, citizen science, eco‐evolutionary feedback, gene flow, landscape genetics, urban evolution, urban socioecology

## Abstract

Urban ecosystems are rapidly expanding throughout the world, but how urban growth affects the evolutionary ecology of species living in urban areas remains largely unknown. Urban ecology has advanced our understanding of how the development of cities and towns change environmental conditions and alter ecological processes and patterns. However, despite decades of research in urban ecology, the extent to which urbanization influences evolutionary and eco‐evolutionary change has received little attention. The nascent field of urban evolutionary ecology seeks to understand how urbanization affects the evolution of populations, and how those evolutionary changes in turn influence the ecological dynamics of populations, communities, and ecosystems. Following a brief history of this emerging field, this Perspective article provides a research agenda and roadmap for future research aimed at advancing our understanding of the interplay between ecology and evolution of urban‐dwelling organisms. We identify six key questions that, if addressed, would significantly increase our understanding of how urbanization influences evolutionary processes. These questions consider how urbanization affects nonadaptive evolution, natural selection, and convergent evolution, in addition to the role of urban environmental heterogeneity on species evolution, and the roles of phenotypic plasticity versus adaptation on species’ abundance in cities. Our final question examines the impact of urbanization on evolutionary diversification. For each of these six questions, we suggest avenues for future research that will help advance the field of urban evolutionary ecology. Lastly, we highlight the importance of integrating urban evolutionary ecology into urban planning, conservation practice, pest management, and public engagement.

## 1 introduction

We are living in a time of unprecedented global change, and there is a pressing need to understand how urbanization affects the evolutionary ecology of life. Urban areas are the fastest growing ecosystem on Earth (United Nations, 2015), with the development of cities leading to changes in many aspects of the environment (Grimm et al., 2008; McKinney, 2006). On average, urban areas experience increased air, water, light, and noise pollution, more impervious surfaces (e.g., buildings and paved roads), greater habitat loss and fragmentation, as well as more non‐native species compared to nearby nonurban habitats (McDonnell, Hahs, & Breuste, 2009; Niemelä, 2011). As such, cities tend to be more similar to one another in many biotic and abiotic environmental characteristics than they are to nearby nonurban ecosystems (Groffman et al., 2014). Urban ecology has provided increasing evidence of how these environmental changes affect species’ population ecology, community structure, and ecosystem processes. However, we know much less about how the ecological impacts of urbanization affect the evolution of populations of organisms living in cities (Donihue & Lambert, 2014; Johnson & Munshi‐South, 2017; Johnson, Thompson, & Saini, 2015), and how this evolution may feedback to influence ecological processes and patterns through eco‐evolutionary dynamics (Alberti, 2015; Hendry, 2016). We refer to this relationship between ecology and evolution in cities as urban evolutionary ecology.

This Perspective article provides a roadmap for future fundamental and applied research in urban evolutionary ecology. We first provide a brief history of the field. We then concisely synthesize current work in urban evolutionary ecology and identify six important unresolved questions that should be addressed to substantially improve our understanding of how urbanization influences evolution. These questions are as follows: (¡) Under what conditions does urbanization affect nonadaptive evolutionary processes? (¡¡) How does urbanization affect natural selection? (¡¡¡) How common are convergent evolutionary responses to urbanization across different species, traits, and genes? (¡v) How does environmental heterogeneity within and among urban landscapes influence evolution? (v) To what extent is a species’ abundance in cities the result of ancestral characteristics, recent adaptation, or phenotypic plasticity? And, (v¡) can urbanization increase diversification, leading to the evolution of novel traits and the origin of species?

In the final section of our paper, we consider how evolution in urban populations may lead to eco‐evolutionary feedbacks with applied implications for ecosystem processes and function. Specifically, we discuss the application of urban evolutionary ecology to urban planning and design, conservation, pest management, and to opportunities for advancing education and public engagement. Overall, this roadmap will facilitate our ability to address fundamental and applied problems in the evolutionary ecology of species living in urban areas.

## 2 a brief history of urban evolutionary ecology

**Figure 1:**
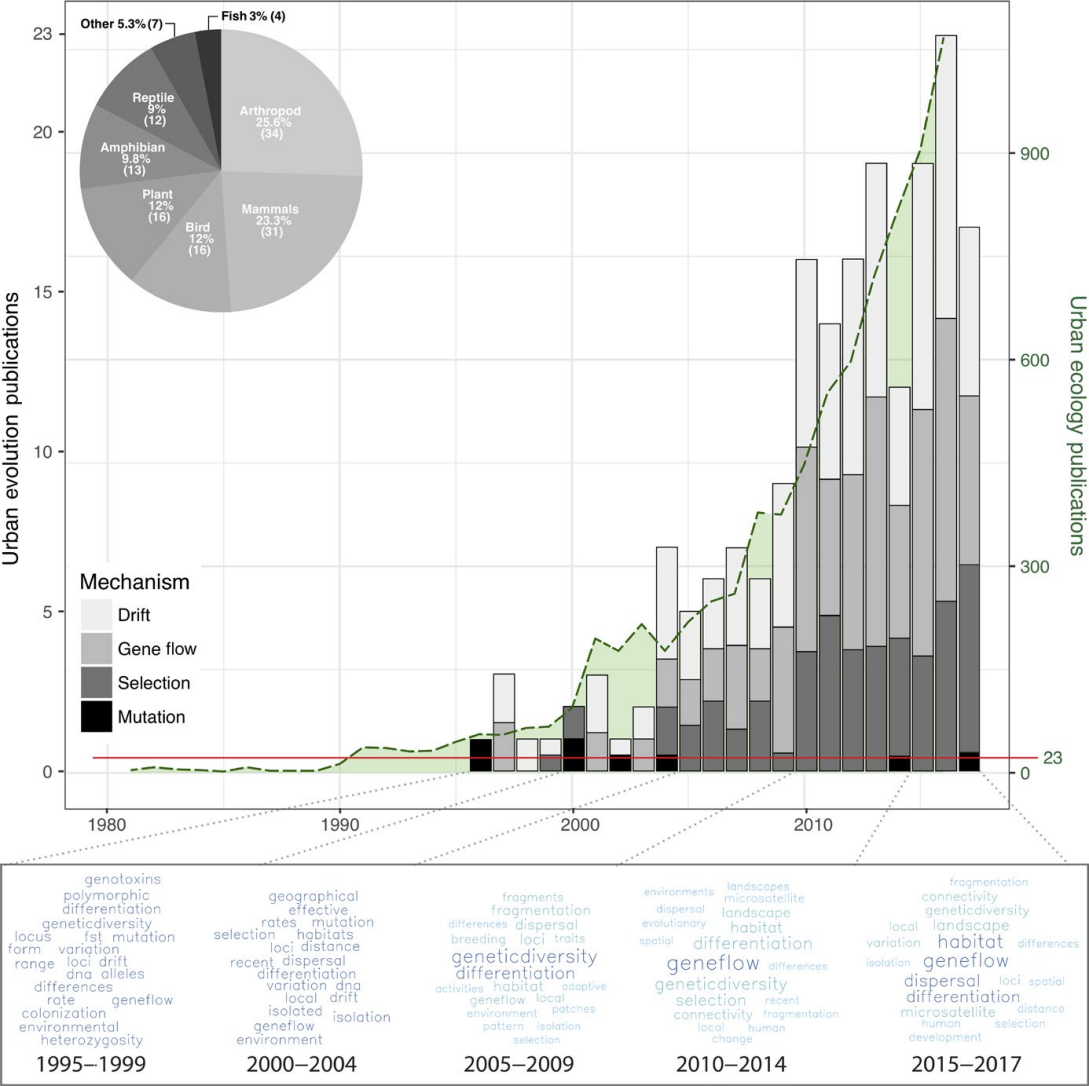
Number of publications (1980–2017) that include the terms “urban ecology” or “urban evolution” (no studies could be found before 1995), combined with word clouds of the most popular keywords in abstracts of urban evolutionary ecology studies from 1995 to 2017. The left axis (black) displays the number of urban evolution publications per year and corresponds to the stacked bars. Each bar is broken down into the proportion of studies which attributed the mechanism of evolution to genetic drift (light gray), gene flow (gray), selection (dark gray), and mutation (black). The right axis (dark green) displays the number of urban ecology publications by year and corresponds to the light green shaded portion with dark green dashed line. The solid red horizontal line indicates the maximum number of urban evolution publications per year relative to urban ecology publications. Inset: proportion of species studied that belong to the taxonomic groups shown; data taken from Supporting Information Table S1 of Johnson and Munshi‐South (2017)

Urban evolutionary ecology has arisen recently and in parallel with urban ecology, which itself is a relatively new discipline. The pioneering urban ecologist Herbert Sukopp (1998) stated that many early ecologists viewed cities as “anti‐life,” and thus felt they were not worthy of study. Because of this perspective, ecologists were reluctant to study cities, and research on urban ecosystems did not take‐off until the 1990s (McDonnell, 2011). The recognition that urban ecology is an important area of study led to a rapid increase in the number of publications on the subject and the emergence of specialized journals on the topic in the early 2000s, with over 1,000 articles published in 2016 alone (Figure 1).

Urban evolutionary ecology and urban ecology are closely linked disciplines because ecological changes in response to urbanization underpin evolutionary change within populations. For example, the fragmentation and degradation of natural habitats in cities frequently reduce the size and increase the isolation of native populations (Faeth & Kane, 1978; Haddad et al., 2015). These ecological processes are expected to increase stochasticity in allele frequencies through genetic drift and founder effects, and decrease the dispersal and movement of alleles (i.e., gene flow) across urban landscapes (Johnson & Munshi‐South, 2017). Changes in the biotic and abiotic environments along urbanization gradients can also alter natural selection and drive adaptive evolution (Donihue & Lambert, 2014). The earliest evidence of evolution in response to urbanization includes some of the first demonstrations of contemporary adaptation in nature. For example, the dark form of the moth *Biston betularia* Linnaeus (peppered moth) increased in frequency in response to elevated industrial pollution around cities from the early 19th to the mid‐20th centuries (Kettlewell, 1955). Despite such classic examples, the systematic and focused study of evolution in urban environments has only recently gained traction (Figure 1), perhaps for the same reasons that urban ecology was long ignored by ecologists.

The number of studies examining evolution in urban environments has risen dramatically since 2010 (Figure 1). The main reason for this recent attention is the recognition that anthropogenic activities are often associated with the fastest rates of evolutionary change (Alberti et al., 2017; Hendry, 2016), and cities can be considered large‐scale, globally replicated “experiments” for examining evolution. Most of the recent studies in urban evolutionary ecology have focused on genetic drift and gene flow, and 83% of these studies examined animals, particularly mammals, arthropods, and birds (Figure 1 inset). Increased research in urban evolutionary ecology will rapidly advance our understanding of the biology of species in cities and will provide new strategies for preserving biodiversity—a key component of creating green, sustainable cities for the future. We consider the emerging research Questions in detail in the following sections.

## 3 key questions and future research in urban evolutionary ecology

Evolutionary ecologists now recognize that cities are living laboratories ideally suited to study evolution. Due to the nascent history of the field and its ongoing rapid development, many questions remain unanswered. We have brought together many of the leading researchers in urban evolution and urban ecology to identify the most important unresolved questions, which represent critical gaps in our knowledge. In this section, we discuss the six major questions we have identified in urban evolutionary ecology, and outline avenues for future research in the field.

### 3.1 Under what conditions does urbanization affect nonadaptive evolutionary processes?

Nonadaptive evolutionary mechanisms include mutation, genetic drift, and gene flow, and each may play important roles in the evolution of populations in urban environments. In considering mutation, several studies show that urban pollution can increase mutation rates in birds and mammals (Somers, McCarry, Malek, & Quinn, 2004; Yauk, Fox, McCarry, & Quinn, 2000). However, these studies used restriction enzyme‐based methods to assess the frequency of new mutations—no study has directly sequenced genomes to identify the frequency and types of de novo mutations induced by urban pollution. As a result, it is unclear if urban pollution increases mutation rates, or the distribution of fitness effects of these mutations. The lack of any answer to such questions is a fundamental gap in our understanding of biology in the Anthropocene, and one with potentially profound consequences. Not only is mutation the ultimate source of genetic variation for all evolution, but if urban pollution elevates mutation rates, then it may have substantial consequences for the health of humans and other organisms, especially if urban‐induced mutations are frequently deleterious as is expected from previous (nonurban) research (Eyre‐Walker & Keightley, 2007). Once this question is answered, we can begin to investigate whether evolution in response to urbanization typically stems from existing standing genetic variation or from novel urban‐induced mutations (Reid et al., 2016; Thompson, Renaudin, & Johnson, 2016; van't Hof, 2016; Wirgin et al., 2011).

**Figure 2:**
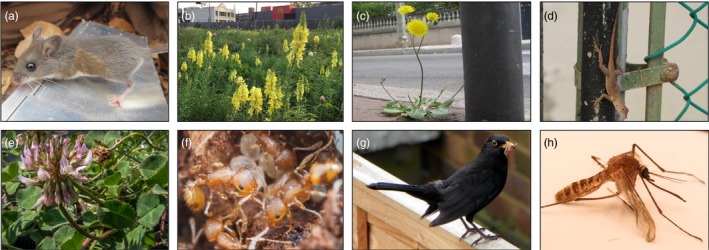
Examples of organisms in which urban evolutionary ecology has been studied. (a) *Peromyscus leucopus* (white‐footed mouse; photo credit: J. Richardson), (b) *Linaria vulgaris* (yellow toadflax; photograph credit: A. Longley), (c)* Crepis sancta* (holy hawksbeard; photograph credit: G. Przetak), (d)* Anolis cristatellus* (crested anole; photograph credit: K. Winchell), (e) *Trifolium repens* (white clover; photograph credit: J. Santangelo), (f)* Temnothorax curvispinosus* (acorn ant; photograph credit: L. Nichols), (g) *Turdus merula *(common blackbird; photograph credit: Wikimedia Commons), and (h) *Culex pipens* f. *molestus *(house mosquito; photograph credit: Wikimedia Commons)

Genetic drift increases in many species as the extent of urbanization increases. For example, fragmented habitats have led to reduced genetic diversity within populations and greater genetic differentiation among urban populations of *Peromyscus leucopus* Rafinesque (white‐footed mice; Figure 2a; Munshi‐South, Zolnik, & Harris, 2016) and *Linaria vulgaris* Mill. (yellow toadflax; Figure 2b; Bartlewicz, Vandepitte, & Jacquemyn, 2015), compared to populations in nonurbanized habitats. However, questions remain unanswered. For example, what types of natural (e.g., rivers and forests) and artificial (e.g., roads and buildings) features of cities affect population demography (e.g., population size) and gene flow to influence genetic diversity within and genetic differentiation among urban populations? Are native species more susceptible to drift in cities than nonnative species, especially when the latter are primarily associated with anthropogenic environments (e.g., synurban or human‐commensal species)? Although the outcome of increased drift (i.e., reduced within‐population diversity) has been identified in multiple cities and species, few studies have examined the demographic processes by which urban development affects historical and contemporary effective population sizes (Lourenço, Alvarez, & Wang, 2017; Ravinet et al., 2018). Addressing these questions will help determine whether genetic drift is more prominent in urban relative to nonurban populations.

Urbanization can have varied effects on gene flow within cities. Gene flow can be restricted by urban features (Beninde, Feldmeier, Veith, & Hochkirch, 2018), relatively unaffected by urbanization (Noreen, Nissalo, Lum, & Webb, 2016), or even enhanced in cities (Björklund, Ruiz, & Senar, 2010). For relatively isolated urban populations, the landscape elements that maintain weak to moderate gene flow are poorly understood. Gene flow can be greatly influenced by the heterogeneity of urban environments even in abundant urban pests, such as *Cimex lectularius* Linnaeus (bed bugs; Booth, Balvín, Vargo, Vilímová, & Schal, 2015), *Rattus norvegicus* Berkenhout (brown rats; Combs, Puckett, Richardson, Mims, & Munshi‐South, 2018), and *Blattella germanica* Linnaeus (German cockroach; Vargo et al., 2014). The extent to which gene flow occurs among populations in different cities is also poorly understood. Humans can facilitate the dispersal of some species (e.g., *Latrodectus hesperus* Chamberlin and Ivie [black widow spiders]) over much longer distances than is typical in nonurban environments (Miles, Dyer, & Verrelli, 2018), and this effect may be enhanced by urban‐adapted phenotypes that promote survival after movement between cities. Other species may colonize cities from nearby surrounding nonurban areas (Evans et al., 2009), and experience selection that further promotes the success of the newly established population (Mueller, Partecke, Hatchwell, Gaston, & Evans, 2013). Future work should aim for large‐scale sampling across multiple urban landscapes, characterize genomewide genetic variation (e.g., Combs et al., 2018; Mueller et al., 2018; Ravinet et al., 2018), and employ statistics in landscape and spatial population genetics to better understand the urban features and traits of organisms that affect rates of gene flow within and between cities (Beninde et al., 2018; Miles, Dyer, et al., 2018; Miles, Johnson, Dyer, & Verrelli, 2018).

### 3.2 How does urbanization affect natural selection?

Cities dramatically alter the abiotic and biotic environment, which may influence natural selection on urban‐dwelling populations. Despite this simple prediction, the agents that impact fitness can be difficult to discern, partly because environmental change in cities ranges from simple to complex. Urbanization includes environmental change along multiple interacting dimensions, such as the amount of impervious surface, temperature, pollution of various forms (e.g., soil, air, water, light, and sound), resource availability, as well as the abundance and diversity of competitors, predators, and mutualists. This multidimensionality can dramatically increase the complexity of environments to which populations must adapt, and can make discovering the agents of selection challenging. Despite these challenges, studies are beginning to uncover features of urban environments that operate as selective agents on resident populations, and how those agents target specific traits (Supporting Information Table S1). For example, one of the first demonstrations of the effects of urban environments on selection was performed in the plant *Crepis sancta* (L.) Babc. (holy hawksbeard; Figure 2c; Cheptou, Carrue, Rouifed, & Cantarel, 2008). This plant has heritable variation for the proportion of dispersing and nondispersing seeds, and experiments showed that urban habitat fragmentation imposes selection for more nondispersing seeds which are more likely to land in substrates suitable for germination in urban habitats. In another example, Winchell and colleagues (Winchell, Carlen, Puente‐Rolón, & Revell, 2018; Winchell, Reynolds, Prado‐Irwin, Puente‐Rolón, & Revell, 2016) discovered that urban populations of the lizard *Anolis cristatellus* Duméril and Bibron (crested anole; Figure 2d) have evolved longer limbs and toe pads with more lamellae in cities of Puerto Rico. Experiments show that these evolved traits increase the locomotor performance of urban lizards on flat, smooth, artificial surfaces commonly found in cities (Winchell, Maayan, Fredette, & Revell, 2018). Together, these examples demonstrate how traits may diverge with urbanization in response to specific urban features. Such clear examples of the effects of urban environments on evolution by natural selection exist in few other urban systems, and thus, this represents an important aspect missing from our understanding of evolution in urban environments.

Identifying generalities about the strength, form, and agents of selection in urban environments would facilitate a mechanistic understanding of adaptive processes associated with urbanization. Rates of phenotypic change are higher in urban environments compared to natural and anthropogenically impacted nonurban habitats (Alberti et al., 2017). This result is consistent with urban environments altering selection on populations, but alternative nonadaptive explanations are also consistent with these patterns (e.g., differences in the influence of genetic drift or phenotypic plasticity), and direct tests of the role of selection have only recently been reported (Santangelo, Rivkin, & Johnson, 2018). It is also unclear whether urban environments impose selection on a small number of ecologically important traits, or whether many traits are selected simultaneously by multiple agents. Existing evidence suggests that selection in cities ranges from relatively simple (i.e., a single agent of selection acting on a single trait: Wirgin et al., 2011; van't Hof et al., 2016) to multifarious (i.e., multiple agents of selection acting on multiple traits: Caizergues, Gregoire, & Charmantier, 2018; Irwin, Warren, & Adler, 2018; Yakub & Tiffin, 2017), but more studies are needed to reach a general conclusion. Identification of the specific features of urban environments that are associated with divergent natural selection will facilitate the interpretation of patterns of convergent evolution (see Section 3.3).

Several empirical approaches will be useful for understanding the role of natural selection in urban environments. We advocate for approaches that estimate phenotypic or genotypic selection using observational and experimental approaches (Lande & Arnold, 1983; Rausher, 1992), combined with genomewide estimates of selection (Schell, 2018). For example, to quantify the strength of natural selection on phenotypes in urban environments, it is necessary to estimate standardized selection gradients and differentials (Kingsolver et al., 2001; Siepielski et al., 2017). These standardized data can then be used to conduct meta‐analyses that contrast natural selection in urban environments with nonurban environments. In addition to selection gradients, more studies that compare genetically based phenotypic divergence and local adaptation between urban and nonurban areas are needed to determine whether divergence across a suite of traits is the norm and whether local adaptation is common. This will require common garden experiments that account for the effects of phenotypic plasticity and identify which trait differences are genetically determined. Complementary field or laboratory experiments (e.g., reciprocal transplant experiments) can test for local adaptation (Gorton, Moeller, & Tiffin, 2018) and characterize the influence of phenotypic plasticity, followed by experiments that manipulate specific environmental variables that have putatively driven adaptive evolution (Brans, Stoks, & De Meester, 2018; Diamond, Chick, Perez, Strickler, & Martin, 2018). Lastly, identifying genomic targets of selection and linking them to phenotypes and environmental features associated with urbanization are necessary to build a mechanistic understanding of how natural selection operates in urban systems (Harris, Munshi‐South, Obergfell, & O'Neill, 2013; Ravinet et al., 2018; Reid et al., 2016; Theodorou et al., 2018).

### 3.3 How common is convergent evolution across different species, traits, and genes?

**Figure 3:**
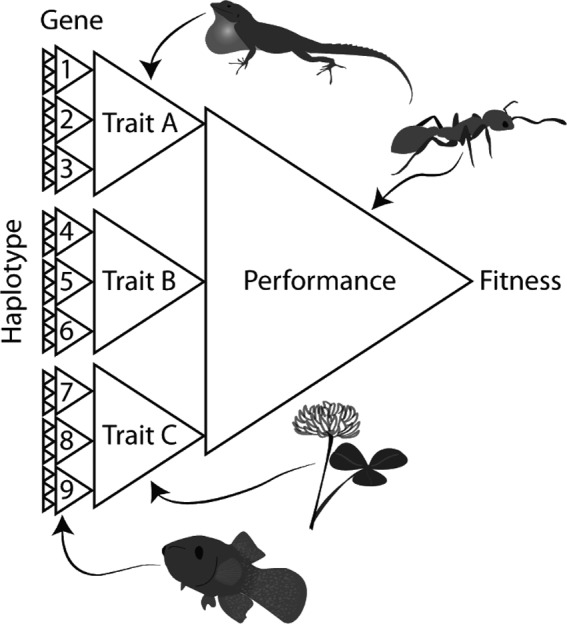
Convergent evolution can occur at phenotypic and genetic levels. At the highest level (organism performance), there are few solutions that would result in high fitness, leading to a large amount of parallel adaptation. But there are several different traits that could aid in that performance and even more potential genetic changes that could result in each trait shift. Each of those genetic changes in turn could be affected through multiple different genotypes. At the lowest level (genetic), many different genotypes can produce the same phenotype, decreasing the probability of observing parallelism at this level. For example, at the gene level: *Fundulus heteroclitus* (mummichog) at polluted sites differed in the same genes (but different haplotypes) related to pollution tolerance (Reid et al., 2016). At the trait level: in *Anolis cristatellus* (crested anole) and *Trifolium repens* (white clover), the same trait changes (morphology in anoles, cyanogenesis in *T. repens*) were observed in multiple urban populations (Thompson et al., 2016; Winchell et al., 2016). At the whole‐organism performance level: *Temnothorax curvispinosus* (acorn ant) exhibited a similar change in thermal performance in multiple urban populations (Diamond et al., 2018)

One of the outstanding questions in evolutionary biology concerns the extent to which taxa occupying similar environments adapt in similar ways (Losos, 2017; Oke, Rolshausen, LeBlond, & Hendry, 2017; Stuart et al., 2017). Here, we refer to independent evolution of similar features as convergent evolution, which research has shown is common at both the phenotypic and genetic levels (Conte, Arnegard, Peichel, & Schluter, 2012; McGhee, 2011; Figure 3). One powerful approach for studying convergent evolution is to experimentally subject replicate populations to similar selective conditions (Lenski, 2017). The shared ecological features of cities enable us to test predictions about the repeatability of evolution (Donihue & Lambert, 2014). Specifically, data from cross‐comparative studies of evolution among cities would allow one to determine: (a) whether populations of the same species undergo convergent evolution in distinct cities; and (b) whether populations of different species undergo convergent evolutionary change in response to similar urban environmental factors.

Empirical studies on adaptation to urban environments show that convergent evolution may be common even among distantly related species (Johnson, Prashad, Lavoignat, & Saini, 2018; Reid et al., 2016; Theodorou et al., 2018; Winchell et al., 2016; Yakub & Tiffin, 2017), yet several questions remain unresolved. First, it is important to understand why convergence is often imperfect (Bolnick, Barrett, Oke, Rennison, & Stuart, 2018), such as the evolution of hydrogen cyanide clines in *Trifolium repens* L. (white clover; Thompson et al., 2016, Figure 2e), heat tolerance in *Temnothorax curvispinosus* Mayr (acorn ant: Diamond et al., 2018, Figure 2f), and the direction of allele frequency changes in a harm‐avoidance gene (*SERT*) of *Turdus merula* Linnaeus (blackbirds; Mueller et al., 2013, Figure 2g). Thus, it is necessary to quantify how often urban environments result in genetic and phenotypic convergence (Oke et al., 2017; Stuart et al., 2017). Second, research should use recently developed statistical frameworks (e.g., Phenotypic Change Vector Analysis, Adams & Collyer, 2009; Bolnick et al., 2018) to quantify the extent to which convergence reflects consistency in the direction of divergence (e.g., urban populations have larger trait values than nonurban populations in some cities but smaller values in others), as well as the magnitude of divergence (i.e., the absolute difference in mean trait values between urban and nonurban populations). Third, it is important to identify the factors that contribute to incidences of nonconvergence. Potential candidates for nonconvergence include variation in selection among cities (Diamond et al., 2018; Thompson et al., 2016), differences in the influence of genetic drift or gene flow among populations (Beninde et al., 2018; Miles, Dyer, et al., 2018; Munshi‐South et al., 2016; Santangelo, Johnson, & Ness, 2018), or the age of cities and thus the amount of time populations have had to adapt to environmental changes (Barnes, Duda, Pybus, & Thomas, 2011).

We suggest several approaches to address gaps in our understanding of the prevalence of convergent evolution among cities. The most important is to study populations and species across replicated urban to nonurban gradients. Replicated designs allow researchers to leverage the power of repeated urbanization for drawing insights into evolutionary convergence. Additionally, insight into the causes of (non)convergence requires the measurement of biotic and abiotic variables experienced by populations. Genetic and genomic approaches can also be implemented to identify gene regions that may have been targets of selection (Ravinet et al., 2018; Reid et al., 2016; van't Hof et al., 2016) and to assess the genetic mechanisms responsible for convergent evolution (Schell, 2018). As with natural selection, convergent local adaptation can be tested with reciprocal transplant experiments between urban and nonurban environments within and among multiple replicated cities. Where reciprocal transplants are infeasible for practical or ethical reasons, field measurements of phenotypic selection (Irwin et al., 2018; Start, Bonner, Weis, & Gilbert, 2018), and laboratory experiments (Brans et al., 2018; Diamond et al., 2018) that manipulate key stressors can generate additional insights into convergent patterns of selection and adaptation. Finally, it is important for studies to test the alternative hypotheses that genetic drift or altered gene flow has led to population differentiation and even parallel evolution in gene frequencies across environments (Colautti & Lau, 2015; Santangelo et al., 2018; Vasemägi, 2006). This can be done through explicit simulation modeling of how genetic drift, gene flow, and natural selection affect evolution, which can also provide predictions about the mechanisms, rate, and likelihood of convergent evolution in response to urbanization (Santangelo et al., 2018).

### 3.4 How does environmental heterogeneity within and among cities influence evolution?

Although cities often share environmental features when averaged across an urban area, they can exhibit considerable spatial and temporal heterogeneity at a finer scale (Niemelä, 2011; Pickett et al., 2017). Cities are a mosaic of habitats that change through time, and over small spatial scales. Consider, for example, how the vegetation, impervious surface, temperature, and noise change as one walks from a large city park to a nearby dense suburb or city center. Variation within and even among cities provides an opportunity to understand how environmental heterogeneity shapes evolutionary processes. Currently, little is known about how such variation within and among cities alters the evolution of species, which represents an important gap in urban evolutionary ecology.

The evolutionary consequences of urban environmental heterogeneity have largely been studied from a population genetics perspective, with a particular emphasis on how habitat fragmentation affects gene flow and genetic drift. For example, several studies have incorporated landscape genetic approaches to identify features of urban landscapes that restrict or facilitate gene flow among urban populations. Munshi‐South (2012) used tree canopy cover, whereas, Unfried, Hauser, and Marzluff (2013) used the age of development and land cover type to investigate altered patterns of gene flow and genetic differentiation among urban populations in *P. leucopus* (Figure 2a) and *Melospiza melodia* Wilson (song sparrows), respectively. Similarly, Beninde et al. (2016; 2018) examined how natural and anthropogenic features in cities affect patterns of gene flow in native, introduced, and hybrid populations of *Podarcis muralis* Laurenti (wall lizard). They found that natural features such as rivers acted as the largest barrier to gene flow, whereas railway lines were a major source of gene flow for hybrid lineages. Environmental variation within cities may also lead to altered natural selection and fine‐scale local adaptation. For example, urban populations of *Ambrosia artemisiifolia* L. (common ragweed) are more phenotypically differentiated from one another than are rural populations, although it remains to be confirmed if this pattern is the result of heterogeneous natural selection (Gorton et al., 2018). These studies highlight the potential importance of combining landscape genetic approaches and experiments with high‐resolution environmental data to understand the effects of environmental heterogeneity within cities for urban evolutionary processes.

Environmental differences among cities can also affect genetic and phenotypic divergence between urban and nonurban populations (Reid et al., 2016; Thompson et al., 2016; Winchell et al., 2016; Yakub & Tiffin, 2017). As mentioned previously, cities can vary in their age, size, human population density, socioeconomic factors, climate, vegetation cover, and the history of development and disturbance (McDonnell et al., 2009; Niemelä, 2011). These differences could affect the evolution of populations in response to urbanization, which may explain some cases of nonconvergent evolution in response to urbanization. For example, the genetic divergence and reproductive isolation between the underground form of *Culex pipiens* Linnaeus f. *molestus* (house mosquito, Figure 2h) and the more typical aboveground form (*C. pipiens *f. *pipiens*) in northern European cities are reportedly absent in more southern cities, possibly due to the absence of diapause in southern populations (Byrne & Nichols, 1999). Similarly, human populations from older cities exhibit a higher frequency of alleles conferring resistance to pathogens like tuberculosis and leprosy than people from younger cities (Barnes et al., 2011). Further research is required to understand how the balance of convergent versus heterogeneous environmental features within and among cities affects evolutionary processes.

### 3.5 To what extent is the success of species in cities the result of ancestral characteristics, rapid adaptation, or phenotypic plasticity?

Phenotypic traits determine the ability of a species to colonize and establish a population in urban environments. It is presently unknown whether traits related to the establishment and proliferation of populations in urban areas are the result of ancestral characteristics, recent adaptation, phenotypic plasticity (including epigenetic change), or a combination of these attributes. Multiple biotic and abiotic processes determine the subset of species present in cities (Aronson et al., 2016; Williams et al., 2009). Species have evolved traits in their historic ranges that might confer an ecological advantage in urban environments. For example, many urban plants and birds native to rocky habitats, such as cliffs, thrive in cities with an abundance of impervious surfaces and vertical structures (Johnston & Janiga, 1995; Lundholm & Marlin, 2006). Alternatively, traits that evolved in response to anthropogenic environments outside of cities might allow a species to be successful in urban habitats (e.g., behavioral plasticity that allows some animals to coexist at high densities in cities; Hulme‐Beaman, Dobney, Cucchi, & Searle, 2016). Some species might also be capable of adapting in just a few generations. Although rapid adaptation in cities has been found for some plant and animal species (Johnson & Munshi‐South, 2017), it is unclear if this is a common phenomenon. Furthermore, the conditions that cities impose on species may select for populations that are phenotypically plastic for one or more traits (Brans et al., 2017; Diamond, Chick, Perez, Strickler, & Martin, 2017), which could enable individuals to colonize, survive, and reproduce across diverse urban habitats (Crispo, 2008). Untangling the relative importance of adaptation versus plasticity for a species’ success in a given city will contribute to understanding how and why some species, but not others, are able to colonize and persist in urban environments (Brans & De Meester, 2018).

Although many urban species exhibit phenotypic plasticity, rarely has it been identified as a key driver of a species’ success in cities (Slabbekoorn, 2013). The role of plasticity cannot be determined unless both the degree of plasticity and the heritability of urban adaptations are explicitly quantified (Brans & De Meester, 2018; Brans et al., 2017; Diamond et al., 2017; Winchell et al., 2016). Common garden experiments and concurrent environmental manipulations are well suited to answer such questions (see Section 3.2 for details). If differences in traits between urban and nonurban populations represent genetic adaptations instead of nonheritable plasticity, then genomic methods can be used to examine signatures of rapid adaptation to urbanization (e.g., selective sweeps: Harris et al., 2013; Ravinet et al., 2018; Theodorou et al., 2018). Gene expression analysis can also be used to identify adaptive divergence and the genetic underpinnings of phenotypically plastic responses to urbanization (Harris, O'Neill, & Munshi‐South, 2015). Furthermore, analyzing patterns of urban trait filtering at the community level will facilitate making inferences about the prevalence of traits that influence the success of species in cities (Knapp et al., 2012). Together these approaches will clarify the relative importance and interactions among ancestral characteristics, rapid adaptation, and phenotypic plasticity in determining the ecological success of urban populations.

### 3.6 Is urbanization driving evolutionary innovation and speciation?

It is well known that urbanization can have negative ecological effects by driving local extinctions, but emerging evidence also suggests that the growth and spread of human populations across the globe, and the ensuing development of cities, are a major cause of contemporary evolutionary diversification. As outlined above, urban populations are often genetically differentiated from nonurban populations, due to both adaptive and nonadaptive evolutionary processes. Because of urban‐driven genetic differentiation, it was recently argued that cities are ideally suited for the study of speciation, and these authors predict that contemporary speciation in cities is ongoing (Thompson, Rieseberg, & Schluter, 2018). This is an important problem, and we propose that it can be generalized into a broader question: Does urbanization lead to evolutionary diversification and innovation of all types, from the origin and spread of new or previously rare alleles and traits, to the origin of species?

Although existing data do not allow for a clear answer, preliminary evidence suggests that humans and cities may be emerging as among the most important drivers of evolutionary innovation in nature. The presence of numerous pest species (e.g., rats, bedbugs, cockroaches, lice) and human commensals (e.g., pigeons, house sparrows, white clover) specifically adapted to living on or around humans indicates that species have already evolved to specialize on the environments that we create (Johnson & Munshi‐South, 2017; Thompson et al., 2018). Recent genomic evidence from *Bombus lapidarius* Linnaeus (red‐tailed bumblebees; Theodorou et al., 2018), *Passer domesticus* Linnaeus (house sparrows; Ravinet et al., 2018), and *Athene cunicularia* Molina (burrowing owls; Mueller et al., 2018) shows that this process of colonization and adaptation to human environments is ongoing. For example, house sparrows originated only 11 Kya, and their adaptation to human environments has involved adaptive evolution of starch metabolism genes, presumably in response to feeding on human‐processed foods (Ravinet et al., 2018). In addition, many bird species show decreased wariness to humans (Møller, 2012), and in some cases, this has been shown to have a genetic basis (Mueller et al., 2013; van Dongen, Robinson, Weston, Mulder, & Guay, 2015). These examples offer emerging evidence that genetic adaptation to urban environments leads to the evolution of novel traits and behaviors.

The question remains, can such divergence lead to speciation? Urban‐driven speciation has only been studied in‐depth in the London Underground Mosquito (*C. pipiens *f. *molestus*), and the existing evidence is consistent with in situ speciation in cities (Byrne & Nichols, 1999). To demonstrate that urbanization drives speciation in other systems, it will be necessary to link urbanization with genetic divergence, barriers to gene flow, and the evolution of reproductive isolation (Thompson et al., 2018). As discussed in Sections 3.1 and 3.2, the first two criteria have been demonstrated by multiple studies; to concretely test for evolutionary divergence, it is now necessary to identify potential mechanisms of reproductive isolation and to quantify the strength of these barriers. We see addressing this gap in our knowledge, including the role of urbanization in driving the evolution of genetic and phenotypic innovation more generally, as among the most important challenges for future research in urban evolutionary ecology.

## 4 applications of urban evolutionary ecology

Understanding how cities affect the evolutionary ecology of species can provide tools to address applied problems related to urban design, conservation, pest management, and education. Knowledge about urban evolution can help inform city planning and design, which can facilitate conservation management to mitigate the negative effects of cities on urban‐dwelling organisms, while finding solutions to control invasive pests. Research in urban evolutionary ecology can also be harnessed to engage and educate the public (Grimm et al., 2008; Lepczyk et al., 2017). In this final section, we highlight how urban evolutionary ecology can be used to address applied problems in our increasingly urbanized planet through the principles of eco‐evolutionary feedbacks (Alberti, 2015; Hendry, 2016).

### 4.1 Urban planning and design

Designing cities that facilitate the coexistence of humans with other organisms is a key priority in an urbanizing world (Nilon et al., 2017). However, traditional practices in both planning and management rely on a view of biodiversity and ecosystem function that is still predominantly static, aiming to maintain current biodiversity, or to achieve preurban conditions (Santamaría & Méndez, 2012). Despite increasing evidence of rapid evolutionary change and its implications for ecosystem function through eco‐evolutionary feedbacks (Alberti, 2015; Hendry, 2016; Rudman, Kreitzman, Chan, & Schluter, 2017), city planners have largely neglected evolutionary processes and the mechanisms that allow species to adapt to novel environmental conditions (Hendry et al., 2010; Moritz & Potter, 2013). For example, in recent decades, cities have increasingly invested resources to implement green infrastructure (e.g., street trees, green roofs and walls, vegetated drainage ditches) to mitigate the ecological impact of urbanization. However, to maintain ecosystem function in a rapidly changing environment, it is important to also facilitate the adaptation of populations to urban habitats, rather than a singular strategy of restoring historic conditions (Olivieri, Tonnabel, Ronce, & Mignot, 2016). This requires incorporating findings from urban evolutionary ecology into planning and design of cities. For example, landscape connectivity of natural or naturalized habitats can facilitate gene flow and prevent the loss of genetic diversity in urban populations (Beninde et al., 2016; Munshi‐South, 2012). Thus, incorporating corridors and remnant natural areas into city planning and design may promote evolutionary processes in cities that maintain the long‐term viability of native species.

Understanding the role that cities play in evolutionary dynamics could prompt city planners to rethink the design of conservation strategies (Alberti, 2015; Olivieri et al., 2016). To achieve this outcome, it will be necessary for urban scientists to redefine how they study urban ecosystems and communicate their findings to help decision‐makers incorporate evolutionary insights into practice. For example, highways severely limit the dispersal of an endangered Australian lizard, *Tympanocryptis pinguicolla* Mitchell (earless dragon), which has resulted in the isolation and genetic differentiation of remnant populations, and declines in abundance (Hoehn, Dimond, Osborne, & Sarre, 2013). Ensuring the resilience of urban populations requires large population sizes to maintain sufficient genetic variation for natural selection to act upon (Sgrò et al., 2010). Consequently, to facilitate movement between populations and promote the maintenance of genetic diversity and long‐term persistence of populations, the authors recommended the protection, rehabilitation, and connection of the lizard's grassland habitats (Hoehn et al., 2013). Large, diverse populations can also be achieved through strategies such as building and conserving sufficiently large parks, curbing urban sprawl, and creating dispersal corridors between populations, potentially along existing roadways and railways (Haddad, 2015), as well as riparian zones (Edge et al., 2017). It also requires standardized metrics of evolutionary change to be used to assess the role of local adaptation on underlying patterns of biodiversity and population health in urban environments (Alberti et al., 2017). For example, identifying the environmental conditions in urban habitats that have the largest fitness effects on species can help city managers determine which aspects of the urban environment to prioritize to maximize species persistence and healthy ecosystems.

### 4.2 Conservation

Understanding the broader ecological and ecosystem‐level applications of urban evolution for native species represents an important priority for future research in urban evolutionary ecology. Integrating eco‐evolutionary dynamics into urban conservation planning could help design strategies that facilitate species’ adaptation to urbanization, improve the forecasting of population declines, and develop appropriate conservation and management plans, as introduced in 4.1 (Alberti, 2015; Olivieri et al., 2016). Along with standard trait‐based assessments of vulnerability to urbanization (Bush et al., 2016), mapping the historical distribution and evolutionary histories of urban‐dwelling species could provide additional insight into their conservation. For example, Mueller et al. (2018) examined burrowing owls, which traditionally inhabited increasingly rare grassland habitats, but have recently established populations in three Argentinian cities. The owls have developed the novel behavior of digging their own burrows, instead of relying on the burrows of other species, which may have facilitated their colonization of urban habitats. In performing whole‐genome sequencing of 137 owls, followed by population genomic analyses, they showed that a small number of individuals had independently colonized each city, and that little gene flow between cities has occurred since the populations were established. These founder events have led to lower genetic diversity within urban compared to nonurban populations, which could constrain adaptive evolution and compromise the ability for the long‐term persistence of urban populations. Continued monitoring of the genetic diversity of the populations, in conjunction with strategies to conserve habitats for breeding and hunting by the owls, could help to maintain the owl populations. Analyses of the genetic basis of the newly acquired burrowing behavior of the urban owls could further provide insights into the evolutionary processes that facilitate successful establishment of native species within cities.

Identifying the drivers of evolution in cities and the evolutionary history of species inhabiting them could be used to build new communities that are resilient to urban stresses and support conservation of biodiversity. For example, standard plant community combinations are used by city planners and urban designers in urban greening initiatives to promote both plant and pollinator conservation (e.g., wild bees; Johnson, Fetters, & Ashman, 2017). The plant species in these communities are selected for their contribution to pollinator foraging, but are also based on traits that improve their survival to urban environmental stressors, such as elevated pollution, salinity, and drought (Dvorak & Volder, 2010). These plant community combinations could be improved by using phylogenetic and experimental data to identify populations that promote stable communities and positive ecosystem services in cities (MacIvor, Cadotte, Livingstone, Lundholm, & Yasui, 2016). We see this being applied to the restoration and conservation of any urban community in two complementary ways. First, seeding populations and communities with elevated intraspecific genetic diversity will prevent inbreeding depression, facilitate adaptation to novel environments, and promote the long‐term stability of populations, communities, and ecosystems through eco‐evolutionary feedbacks (Hughes, Inouye, Johnson, Underwood, & Vellend, 2008). Second, monitoring organisms of conservation concern in remnant or restored urban patches and collecting offspring for propagation under similar conditions could create an urban‐adapted stock for future restoration efforts. For example, the Swiss meadow orchid communities remaining on 100+ year old green roofs at the Moos Water Treatment facility in Switzerland provide habitat for rare orchid species, and the seeds of these plants are now used in community restoration around Zürich (Rowe, 2015). This latter case represents one example of the successful integration of urban evolutionary ecology and conservation and could be used as a model for other urban conservation efforts.

### 4.3 Urban pests

Evolutionary analyses have played a role in urban pest control for decades, particularly in understanding and managing the genetic basis of pesticide resistance in bed bugs, cockroaches, lice, rats, and mice (reviewed in Johnson & Munshi‐South, 2017). Commensal rodents in particular have served as a model for understanding the efficacy of pest control in urban environments, including the broader ecological effects of such control measures. Municipal governments have been pressured to reduce the use of anticoagulant rodenticides that secondarily poison urban predators such as raptors. Thus, we expect new evolutionary adaptations in commensal rodents resulting from an evolutionary arms‐race between rodent pests and pesticides. This outcome has already been observed in the case of resistance to warfarin (Rost et al., 2009), but may also be problematic with new control methods. For example, many cities have conducted experimental trials of chemical sterilization baits that operate on both males and females (Dyer & Mayer, 2014). Such strong selection acting directly on reproduction could favor individuals with heritable bait aversion and/or biochemical resistance to the compound.

Population genetic analyses have also played a role in understanding commensal rodent movements and spatial extent of rat populations, with many potential applications to better target pest management efforts. For example, recent analyses of rodent populations in multiple cities have clarified the spatial extent of relatedness and dispersal distances of rats, and identified potential barriers or filters to rat gene flow (Combs et al., 2018; Desvars‐Larrive et al., 2018). Future comparative work across cities should seek to understand how rat movements, interactions with native rodent species, and pest management strategies affect not only evolution in rats, but also the diversity and evolution of known and potentially emerging human pathogens, which are remarkably diverse and a potential threat to human health (Firth et al., 2014; Lee et al., 2018; Williams et al., 2018). These types of considerations represent an important avenue for future research in not just rodents, but any urban pest, such as black widow spiders (Miles, Dyer, et al., 2018), mosquitoes (Byrne & Nichols, 1999), invasive plants (Arredondo, Marchini, & Cruzan, 2018), and others. Productive collaborations between evolutionary ecologists, the pest management industry, and public health agencies are needed to predict and manage inevitable selection pressures caused by pest management in cities and human borne diseases.

### 4.4 Education and community engagement

**Figure 4:**
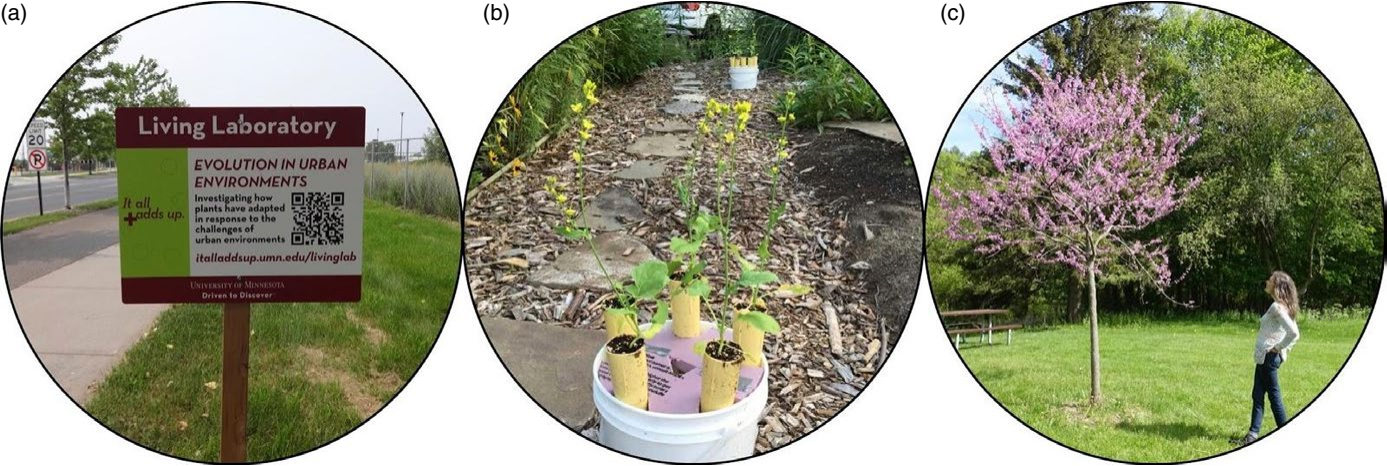
Studies in urban evolutionary ecology offer opportunities to engage the public. The level of engagement can range from passive learning (a), to the donation of private lands for experiments (b), to active community participation in data collection (c). The images show three examples of how authors of the present article have included the community in their past research. (a) Passive learning: Gorton used signs with QR codes linking to the project website to educate the public about evolution in urban environments. (b) Use of private land: Rivkin used yard space provided by homeowners in the Greater Toronto Area to conduct “backyard evolution” experiments. (c) Community science: de Keyzer recruited community scientists to collect spatially extensive phenology data across the large urban area of Toronto, Canada

Urban evolutionary ecology offers opportunities for public engagement and education on the importance of science and evolution for the conservation of native species and mitigation of invasive pests. Education and community engagement can occur through both passive and active methods. Marking study sites with educational signage and providing further information via project websites are two ways scientists can passively engage the public while conducting urban evolutionary ecology studies (Figure 4a). Actively engaging the public in the formulation of research questions and hypotheses, data collection, and the interpretation of results (i.e., “community science,” often called “citizen science”) can provide additional benefits to both researchers and participants. Community engagement can provide opportunities for researchers to access private land to conduct studies (e.g., backyard evolution experiments; Figure 4b) and increase sample sizes through data collected by many individuals (Figure 4c). Benefits to the community include greater appreciation of the scientific process and a better understanding of biological concepts and methods that may be viewed by some as esoteric or even controversial (e.g., evolution and climate change; Yoho & Vanmali, 2016). Community science projects in urban settings can also offer accessible and affordable opportunities for participants to learn about, interact with, and appreciate nature.

Many types of ecological data have been collected by community scientists in urban areas, including biodiversity sampling (e.g., BioBlitz™), tracking invasive (e.g., Ontario's Invading Species Awareness Program, http://www.eddmaps.org/ontario) or at‐risk species (e.g., Monarch Watch, http://www.monarchwatch.org), and monitoring phenological events (e.g., National Phenology Network, https://www.usanpn.org). Data collected by the public have also been used to answer questions in urban evolutionary ecology. In 1983–1984, university biology students collected peppered moths across the UK, which provided evidence for declines in the frequency of the darker morphs in response to decreased postindustrial atmospheric pollution (Cook, Mani, & Varley, 1986). Recently, the Evolution MegaLab project recruited the public to collect data on shell polymorphisms in *Cepaea nemoralis* L. (brown‐lipped banded snail), which, when compared to historical records, showed evidence of continental‐scale evolutionary change (Silvertown et al., 2011). The newly launched Global Urban Evolution project (http://www.globalurbanevolution.com/) has recruited over 550 scientists and students internationally to collaboratively study convergent evolution in *T. repens* in response to urbanization. These examples demonstrate the reciprocal benefits of community engagement by urban evolutionary ecologists and the usefulness of community scientists in collecting data for large‐scale projects.

## 5 conclusions

The nascent discipline of urban evolutionary ecology is advancing rapidly, and numerous examples of evolutionary change in response to urbanization have been documented. In this Perspective article, we provide a roadmap for future research on urban evolutionary ecology. We highlight six major questions in the field, which if addressed, would greatly advance our understanding of how urbanization affects the evolution of populations. These questions include understanding how urbanization affects: (¡) nonadaptive evolutionary processes, (¡¡) natural selection, (¡¡¡) convergent evolution, (¡v) the influence of environmental heterogeneity on evolution, (v) the roles of plasticity, ancestral traits, and contemporary adaptation for the ecological success of urban species, and finally (v¡) the evolutionary diversification of novel traits, genes, and species. Of equal importance will be to apply the insights gained from urban evolutionary ecology to city planning, conservation, pest management, and public engagement. By integrating information concerning rapid adaptation, dispersal patterns, and the impact of habitat heterogeneity into city management, it may be possible to mitigate the detrimental effects of cities on biodiversity through eco‐evolutionary feedbacks (Alberti, 2015; Hendry, 2016).

## CONFLICT OF INTEREST

None declared.

## DATA ARCHIVING

There are no data associated with this article.
