# Supplementary material for: A roadmap for urban evolutionary ecology
Source: Evol Appl. 2018 Dec 14;12(3):384–98. doi: 10.1111/eva.12734 (PMC6383741; doi:10.1111/eva.12734)
Supplement: Supplementary file 1 [file EVA-12-384-s001.pdf]

Supporting Information

Article title: A roadmap for urban evolutionary ecology.

Table S1. List of documented agents of selection associated with urbanization[1].

Causal 'agent' of selection	Change in urban environment	Evolutionary or selective outcome	Test species	Experiment (E) or Observation (O)[2]	Reference
Altered biotic interactions	Reduced herbivory	Urban populations tend to have reduced extra-floral nectaries	Partridge pea (*Chamaecrista fasciculata*)	O	Rios et al. (2008)
	Different predator community	Selection for more off-ground nests; no evolution documented.	Dark-eyed junco (*Junco hyemalis*)	O	Yeh et al. (2007)
	Increased florivory<br>Greater pollen limitation	Floral characters evolve to reduce florivory.<br>Selection for more flowers	Carolina jasmine (*Gelsemium sempervirens*)	O	Irwin et al. (2014)<br>Irwin et al. (2018)
	Loss of high trophic-level species	Directional selection for smaller galls	Goldenrod gall fly (*Eurosta solidaginus*)	O	Start et al. (2018)
Artificial light	More artificial light	Urban populations are less attracted to light	Small ermine moth (*Yponomeuta cagnagella*)	E & O	Altermatt & Ebert (2016)
Direct interactions with humans	More frequent interactions with humans	Populations evolve reduced wariness of humans	Black swan (*Cygnus atratus*)	E & O	van Dongen et al. (2015)
Habitat fragmentation	Suitable habitat patches separated by impervious surface	Populations evolve lower dispersal	Holy hawksbeard (*Crepis sancta*)	E & O	Cheptou et al. (2008)
	Suitable habitat patches separated by impervious surface	Natural and sexual selection for flight endurance	Azure damselfly (*Coenagrion puella*)	O	Tüzün et al., (2017a)
Increased stress	Elevated perturbations (i.e. noise, human interactions)	Urban birds have reduced stress responses	European blackbird (*Turdus merula*)	O	Partecke et al. (2006)
Novel habitat	Access to 'underground' habitat	Multivariate life histories and behavioural divergence	Common house mosquito (*Culex pipiens* complex)	O	Byrne & Nichols (1999)
	Fewer trees and more smooth, artificial, surfaces	Urban populations evolve longer limbs and more toe lamellae	Crested anole (*Anolis cristatellus*)	O	Winchell et al. (2016, 2018)
Pollution	Urban surfaces covered in dark soot	Dark colour morph rises in frequency	Peppered moth (*Biston betularia*)	E & O	Kettlewell (1955)
	Increased pentachlorobiphenyl (PCB) pollution	Populations adapt to tolerate PCBs	Atlantic tomcod (*Microgadus tomcod*)	O	Wirgin et al. (2011)
	Increased pentachlorobiphenyl (PCB) pollution	Populations adapt to tolerate PCBs	Atlantic killifish (*Fundulus heteroclitus*)	O	Reid et al. (2016)
	Increased lead and zinc pollution	Selection for darker pigmentation in urban areas	Feral pigeon (*Columbia livia*)	E & O	Chatelain et al. (2016)
	Increased toxic pollution	Adaptive evolution of detoxification enzymes	African malaria mosquito (*Anopheles gambiae* complex)	O	Kamdem et al. (2017)
Supplementary human food	Food supplied during winter	Populations adapt to be less migratory (may also be caused by warmer microclimate)	European blackbird (*Turdus merula*)	O	Partecke & Gwinner 2007)
	Novel food items	Relaxed selection on male colouration	Northern cardinal (*Cardinalis cardinalis*)	O	Jones et al., (2010)

	Novel food items	Bimodality in beak shape lost, possibly due to relaxed selection	Medium ground finch (*Geospiza fortis*)	O	de León et al. (2011)
	Novel food items	Evolution of genes involved in metabolic processes	White-footed mouse (*Peromyscus leucopus*)	O	Harris & Munshi-South (2017)
	More bird feeders	Larger beaks evolve in urban areas	House finch (*Carpodacus mexicanus*)	E & O	Badyaev et al. (2008)
	More bird feeders	Larger beaks evolve in urban areas	Great tit (*Parus major*)	O	Bosse et al. (2017)
Temperature	Increased temperature	Urban populations of two of four species have faster growth at higher temperatures	Four species of chytinolytic fungi	E	McLean et al. (2005)
	Increased temperature	Urban populations evolve high heat tolerance	Water flea (*Daphnia magna*)	E & O	Brans et al. (2017, 2018)
	Increased temperature	Urban populations evolve higher critical thermal maximum and minimum	Acorn ant (*Temnothorax curvispinosus*)	E & O	Diamond et al. (2017, 2018)
	Increased temperature	Urban populations grow more quickly under warm temperatures	Azure damselfly (*Coenagrion puella*)	E & O	Tüzün et al. (2017b)
	Colder ground temperatures during winter	Urban populations evolve reduced cyanogenesis	White clover (*Trifolium repens*)	O	Thompson et al. (2016)
Water velocity	Faster flowing water in urban streams	Urban fish populations evolved more streamlines bodies	Blacknose dace (*Rhinichthys obtusus*)	E & O	Kern et al. (2018)
		Urban fish populations evolved deeper bodies	Creek chub (*Semotilus atromaculatus*)		

[1]We omit cases where organisms adapt in response to targeted human removal (e.g. pesticide resistance).

[2]Observational studies document the proposed environmental change between urban and non-urban areas, and experimental studies investigate how the putative agent of selection impacts fitness.

References

Altermatt, F., & Ebert, D. (2016). Reduced flight-to-light behaviour of moth populations exposed to long-term urban light pollution. *Biology Letters*, *12*, 1–4. https://doi.org/10.1098/rsbl.2016.0111

Badyaev, A. V., Young, R. L., Oh, K. P., & Addison C. (2008). Evolution on a local scale: Developmental, functional, and genetic bases of divergence in bill form and associated changes in song structure between adjacent habitats. *Evolution*, *62*, 1951–1964. https://doi.org/10.1111/j.1558-5646.2008.00428.x

Brans, K. I., Jansen, M., Vanoverbeke, J., Tüzün, N., Stoks, R., & De Meester, L. (2017). The heat is on: Genetic adaptation to urbanization mediated by thermal tolerance and body size. *Global Change Biology*, *23*, 5218-5227. http://doi.org/10.1111/gcb.13784

Brans, K. I., Stoks, R., & De Meester, L. (2018). Urbanization drives genetic differentiation in physiology and structures the evolution of pace-of-life syndromes in the water flea *Daphnia magna*. *Proceedings of the Royal Society B: Biological Sciences*, *285*, 20180169. http://doi.org/10.1098/rspb.2018.0169

Bosse, M., Spurgin, L. G., Laine, V. N., Cole, E. F., Firth, J. A., Gienapp, P., ..., Slate, J. (2017). Recent natural selection causes adaptive evolution of an avian polygenic trait. *Science*, *358*, 365–368. https://doi.org/10.1126/science.aal3298

Byrne, K., & Nichols, R. A. (1999). *Culex pipiens* in London Underground tunnels: Differentiation between surface and subterranean populations. *Heredity*, *82*, 7–15. http://doi.org/10.1038/sj.hdy.6884120

Chatelain, M., Gasparini, J., & Frantz, A. (2016). Do trace metals select for darker birds in urban areas? An experimental exposure to lead and zinc. *Global Change Biology*, *22*, 2380–2391. https://doi.org/10.1111/gcb.13170

Cheptou, P.-O., Carrue, O., Rouifed, S., & Cantarel, A. (2008). Rapid evolution of seed dispersal in an urban environment in the weed *Crepis sancta*. *Proceedings of the National Academy of Sciences of the United States of America*, *105*, 3796–3799. http://doi.org/10.1073/pnas.0708446105

Diamond, S. E., Chick, L., Perez, A., Strickler, S. A., & Martin, R. A. (2017). Rapid evolution of ant thermal tolerance across an urban-rural temperature cline. *Biological Journal of the Linnean Society*, *121*, 248–257. http://doi.org/10.1093/biolinnean/blw047

Diamond, S. E., Chick, L. D., Perez, A., Strickler, S. A., & Martin, R. A. (2018). Evolution of thermal tolerance and its fitness consequences: Parallel and non-parallel responses to urban heat islands across three cities. *Proceedings of the Royal Society B: Biological Sciences*, *285*, 20180036. http://doi.org/10.1098/rspb.2018.0036

de León, L. F., Raeymaekers, J. A. M., Bermingham, E., Podos, J., Herrel, A., & Hendry, A. P. (2011). Exploring possible human influences on the evolution of Darwin's finches. *Evolution*, *65*, 2258–2272. https://doi.org/10.1111/j.1558-5646.2011.01297.x

Harris, S.E., & Munshi-South, J. (2017). Signatures of positive selection and local adaptation to urbanization in white-footed mice (*Peromyscus leucopus*). *Molecular Ecology*, *26*, 6336–6350. https://doi.org/10.1111/mec.14369

Irwin, R. E., Warren, P. S., & Adler, L. S. (2018). Phenotypic selection on floral traits in an urban landscape. *Proceedings of the Royal Society B: Biological Sciences*, *285*, 20181239. http://doi.org/10.1098/rspb.2018.1239

Irwin, R. E., Warren, P. S., Carper, A. L., & Adler, L. S. (2014). Plant-animal interactions in suburban environments: Implications for floral evolution. *Oecologia*, *174*, 803–815. https://doi.org/10.1007/s00442-013-2797-2

Jones, T. M., Rodewald, A. D., & Shustack, D. P. (2010). Variation in plumage coloration of northern cardinals in urbanizing landscapes. *Wilson Journal of Ornithology*, *122*, 326–333. http://doi.org/10.1676/09-082.1

Kamdem, C., Fouet, C., Gamez, S., & White, B. J. (2017). Pollutants and insecticides drive local adaptation in African malaria mosquitoes. *Molecular Biology and Evolution*, *34*, 1261–1275. https://doi.org/10.1093/molbev/msx087

Kern, E. M., & Langerhans, R. B. (2018). Urbanization drives contemporary evolution in stream fish. *Global Change Biology*, *24*, 3791-3803. https://doi.org/10.1111/gcb.14115

Kettlewell, H. B. D. (1955). Selection experiments on industrial melanism in the Lepidoptera. *Heredity*, *9*, 323–342. http://doi.org/10.1038/hdy.1955.36

McLean, M. A., Angilletta, M. J., & Williams, K. S. (2005). If you can't stand the heat, stay out of the city: Thermal reaction norms of chitinolytic fungi in an urban heat island. *Journal of Thermal Biology*, *30*, 384–391. https://doi.org/10.1016/j.jtherbio.2005.03.002

Partecke, J., & Gwinner, E. (2007). Increased sedentariness in European blackbirds following urbanization: A consequence of local adaptation? *Ecology*, *88*, 882–890.

https://doi.org/10.1890/06-1105

Partecke, J., Schwabl, I., & Gwinner, E. (2006). Stress and the city: Urbanization and its effects on the stress physiology in European Blackbirds. *Ecology*, *87*, 1945–1952. https://doi.org/10.1890/0012-9658(2006)87[1945:SATCUA]2.0.CO;2

Reid, N. M., Proestou, D. A., Clark, B. W., Warren, W. C., Colbourne, J. K., Shaw, J. R., … Whitehead, A. (2016). The genomic landscape of rapid repeated evolutionary adaptation to toxic pollution in wild fish. *Science*, *354*, 1305–1308. http://doi.org/10.1126/science.aah4993

Rios, R. S., Marquis, R. J., & Flunker, J. C. (2008). Population variation in plant traits associated with ant attraction and herbivory in *Chamaecrista fasciculata* (Fabaceae). *Oecologia 156*, 577–588. https://doi.org/10.1007/s00442-008-1024-z

Start, D., Bonner, C., Weis, A. E., & Gilbert, B. (2018). Consumer-resource interactions along urbanization gradients drive natural selection. *Evolution*, *72*, 1863-1873. https://doi.org/10.1111/evo.13544

Thompson, K. A., Renaudin, M., & Johnson, M. T. J. (2016). Urbanization drives the evolution of parallel clines in plant populations. *Proceedings of the Royal Society B: Biological Sciences*, *283*, 20162180. http://doi.org/10.1098/rspb.2016.2180

Tüzün, N, Op de Beeck, L., & Stoks, R. (2017a). Sexual selection reinforces a higher flight endurance in urban damselflies. *Evolutionary Applications 10*, 694–703. https://doi.org/10.1111/eva.12485

Tüzün, N., Op de Beeck, L., Brans, K. I., Janssens, L., & Stoks, R. (2017b). Microgeographic differentiation in thermal performance curves between rural and urban populations of an aquatic insect. *Evolutionary Applications 10*, 1–9. https://doi.org/10.1111/eva.12512

van Dongen, W. F. D., Robinson, R. W., Weston, M. A., Mulder, R. A., & Guay, P.-J. (2015). Variation at the DRD4 locus is associated with wariness and local site selection in urban black swans. *BMC Evolutionary Biology 15*, 253. https://doi.org/10.1186/s12862-015-05338

Winchell, K. M., Maayan, I., Fredette, J. R., & Revell, L. J. (2018). Linking locomotor performance to morphological shifts in urban lizards. *Proceedings of the Royal Society B: Biological Sciences*, *285*, 20180229. http://doi.org/10.1098/rspb.2018.0229

Winchell, K. M., Reynolds, R. G., Prado-Irwin, S. R., Puente-Rolón, A. R., & Revell, L. J. (2016). Phenotypic shifts in urban areas in the tropical lizard *Anolis cristatellus*. *Evolution*,

70, 1009–1022. http://doi.org/10.1111/evo.12925

Wirgin, I., Roy, N. K., Loftus, M., Chambers, R. C., Franks, D. G., & Hahn, M. E. (2011). Mechanistic basis of resistance to PCBs in Atlantic tomcod from the Hudson River. *Science*, *331*, 1322-1325. http://doi.org/10.1126/science.1197296

Yeh, P. J., Hauber, M. E., & Price, T. D. (2007). Alternative nesting behaviours following colonisation of a novel environment by a passerine bird. *Oikos 116*, 1473–1480. https://doi.org/10.1111/j.0030-1299.2007.15910.x
